# Supplementary material for: Feasibility and Outcomes of the Early Start Denver Model Delivered within the Public Health System of the Friuli Venezia Giulia Italian Region
Source: Brain Sci. 2021 Sep 10;11(9):1191. doi: 10.3390/brainsci11091191 (PMC8464931; doi:10.3390/brainsci11091191)
Supplement: Supplementary file 1 [file brainsci-11-01191-s001.zip › brainsci-1244038-supplementary.pdf]

Table S1. Summary of results for ESDM and TAU groups

	T0-T1		T1-T2		Time		Group		Time x Group	
	Mean Δ ESDM (*N*=19)	Mean Δ TAU (*N*=19)	Mean Δ ESDM (*N*=19)	Mean Δ TAU (*N*=19)	*p*	η_p^2	*p*	η_p^2	*p*	η_p^2
**Development Profile**										
Cognitive	6.68	2.63	2.95	-1.37	**0.013***	*0.11*	*0.957*	*0.01*	*0.106*	*0.06*
Language	6.79	4.37	3.74	5.37	***<0.001****	*0.34*	*0.442*	*0.02*	*0.769*	*0.01*
**ADOS-2**										
CSS SA	-2.16	-1.00	-1.16	-1.16	***<0.001****	*0.46*	***0.048****	*0.10*	*0.173*	*0.05*
CSS RRB	0.53	0.89	0.21	-0.58	*0.091*	*0.06*	***0.034****	*0.12*	*0.494*	*0.02*
CSS Total	-1.37	-0.74	-0.95	-1.00	***<0.001****	*0.35*	*0.378*	*0.02*	*0.560*	*0.02*
**VABS-II**										
Communication	3.46	1.53	4.00	2.84	***0.019****	*0.11*	*0.221*	*0.04*	*0.767*	*0.01*
Daily living skills	-1.07	-2.79	-4.26	-3.16	*0.058*	*0.08*	***0.011****	*0.18*	*0.933*	*0.01*
Socialization	2.07	0.37	-4.16	-1.11	*0.385*	*0.03*	*0.252*	*0.04*	*0.696*	*0.01*
Compound scale	3.19	-6.32	-8.00	-5.89	***0.025****	*0.10*	*0.087*	*0.08*	*0.296*	*0.03*
**ESDM Curriculm Check List**										
Receptive Communication	21.37	13.07	12.95	12.49	***<0.001****	*0.67*	*0.113*	*0.07*	***0.031****	*0.10*
Expressive Communication	18.53	10.57	16.32	11.40	***<0.001****	*0.59*	*0.093*	*0.08*	***0.006****	*0.14*
Joint Attention	5.68	4.53	2.74	3.53	***<0.001****	*0.55*	*0.646*	*0.01*	*0.484*	*0.02*
Social skills	19.37	10.51	11.63	11.70	***<0.001****	*0.51*	*0.113*	*0.07*	***0.049****	*0.09*
Imitation skills	6.26	3.50	5.84	4.43	***<0.001****	*0.50*	***0.009****	*0.19*	***0.039****	*0.09*
Cognitive	6.89	5.46	7.47	6.73	***<0.001****	*0.53*	*0.157*	*0.06*	*0.278*	*0.04*
Play	12.32	6.02	6.79	9.49	***<0.001****	*0.61*	*0.108*	*0.08*	*0.110*	*0.06*
Fine Motor skills	16.95	13.61	7.53	10.27	***<0.001****	*0.61*	*0.432*	*0.02*	*0.598*	*0.01*
Gross Motor skills	7.16	5.02	5.05	3.96	***<0.001****	*0.48*	*0.962*	*0.01*	*0.130*	*0.06*
Adaptive behavior skills	2.32	0.90	0.47	0.96	***0<.001****	*0.24*	***0.031****	*0.13*	*0.439*	*0.02*
Autonomy	15.74	14.63	13.05	21.76	***<0.001****	*0.41*	*0.264*	*0.04*	*0.920*	*0.01*
Level	132.58	89.59	89.84	96.72	***<0.001****	*0.64*	*0.288*	*0.03*	*0.141*	*0.06*
Overall communication	39.89	23.64	29.26	23.89	***<0.001****	*0.66*	*0.094*	*0.08*	***0.008****	*0.14*
Overall social	31.32	18.54	20.21	18.55	***<0.001****	*0.57*	*0.077*	*0.09*	***0.035****	*0.10*
Overall cognitive-play	19.21	10.59	13.74	16.22	***<0.001****	*0.60*	*0.117*	*0.07*	*0.177*	*0.05*
Overall motor	23.68	18.63	13.26	14.23	***<0.001****	*0.62*	*0.611*	*0.01*	*0.361*	*0.03*
Communication, social, cognitive-play and motor	110.58	57.35	74.58	72.89	***<0.001****	*0.62*	*0.180*	*0.05*	***0.015****	*0.12*
**BOSCC**										

Social communication	-4.26	-2.28	-5.63	-2.92	***<0.001****	*0.37*	*0.314*	*0.03*	*0.270*	*0.04*
Restrictive and repetitive behaviors	0.93	-1.83	-0.66	1.87	*0.656*	*0.01*	*0.723*	*0.01*	*0.361*	*0.02*
Total	-3.58	-4.13	-6.29	-1.03	***<0.001****	*0.28*	*0.378*	*0.03*	*0.365*	*0.03*
